# The Measurement of Spatiotemporal Parameters in Running at Different Velocities: A Comparison Between a GPS Unit and an Infrared Mat

**DOI:** 10.3390/mps7060103

**Published:** 2024-12-20

**Authors:** Thomas Provot, Benjamin Millot, Eline Hazotte, Thomas Rousseau, Jean Slawinski

**Affiliations:** 1 EPF–Engineering School, 55 Avenue du Président Wilson, 94230 Cachan, France; thomas.provot@epf.fr (T.P.); eline.hazotte@etudiant.univ-reims.fr (E.H.); thomas.rousseau@etudiant.univ-reims.fr (T.R.); 2 Arts et Métiers Institute of Technology, Institut de Biomécanique Humaine Georges Charpak, IBHGC, UR 4494, 75013 Paris, France, Université Sorbonne Paris Nord, 93000 Bobigny, France; 3 French Athletics Federation (FFA), 75013 Paris, France; benjamin.millot@athle.fr; 4 Laboratory Sport, Expertise and Performance (EA 7370), French Institute of Sport (INSEP), 75012 Paris, France

**Keywords:** GPS, IMU, step frequency, step length, sprinting, coaching

## Abstract

The accurate measurement of spatiotemporal parameters, such as step length and step frequency, is crucial for analyzing running and sprinting performance. Traditional methods like video analysis and force platforms are either time consuming or limited in scope, prompting the need for more efficient technologies. This study evaluates the effectiveness of a commercial Global Positioning System (GPS) unit integrated with an Inertial Measurement Unit (IMU) in capturing these parameters during sprints at varying velocities. Five experienced male runners performed six 40 m sprints at three velocity conditions (S: Slow, M: Medium, F: Fast) while equipped with a GPS-IMU system and an optical system as the gold standard reference. A total of 398 steps were analyzed for this study. Step frequency, step length and step velocity were extracted and compared using statistical methods, including the coefficient of determination (r^2^) and root mean square error (RMSE). Results indicated a very large agreement between the embedded system and the reference system, for the step frequency (r^2^ = 0.92, RMSE = 0.14 Hz), for the step length (r^2^ = 0.91, RMSE = 0.07 m) and the step velocity (r^2^ = 0.99, RMSE = 0.17 m/s). The GPS-IMU system accurately measured spatiotemporal parameters across different running velocities, demonstrating low relative errors and high precision. This study demonstrates that GPS-IMU systems can provide comprehensive spatiotemporal data, making them valuable for both training and competition. The integration of these technologies offers practical benefits, helping coaches better understand and enhance running performance. Future improvements in sample rate acquisition GPS-IMU technology could further increase measurement accuracy and expand its application in elite sports.

## 1. Introduction

In running and sprinting, the step velocity determines the final performance. This step velocity can be defined as the product of the step length and the step frequency, with the latter further determined by the contact times and flight times [1]:

(1) SV=SL·SF

(2) SF=1/(Tc+Tf)

Hence, an increase in the velocity can result from endless combinations of increase in either one (if the other parameter does not undergo a concomitant similar or larger decrease) or both parameters. For instance, in maximal effort accelerated sprinting, the step frequency increases from the first stance to the ~third stance [2,3] and plateaus afterwards, while the step length continues to increase until the maximal velocity (Vmax) [2]. Hence, in over-ground sprinting with maximal effort, the increasing velocity almost exclusively results from an increase in a step length above 7 m/s [2]. In contrast, when running with a constant velocity below 7 m/s, a change in the velocity results from a modification in both the step frequency and the step length. Above 7 m/s, changes in the velocity only result from changes in step length [4,5]. Fatigue also influences the step length, step frequency, contact times and flight times, eventually modifying the velocity [6,7,8]. Thus, measuring the spatiotemporal parameters in running or sprinting during trainings and competitions is a key factor for coaches to understand the underlying parameters that explain the changes in velocity.

To measure these spatiotemporal parameters, different technologies can be used. One of the most popular technologies used by coaches is video analysis. The video analysis permits to directly evaluate the contact times and the flight times, by computing the time duration (i.e., the number of frames) for each event and thus calculate the step frequency (Equation (2)). Using video analysis, it is also possible to compute the step length, yet, for this purpose, the camera must be fixed and the area of measurement calibrated [8,9]. With this technology, the measurements’ accuracy mainly relies on the sampling frequency, the quality of the images captured and the calibration process. Although easily accessible to coaches today, thanks to the integration of high-quality cameras in smartphones, computing the spatiotemporal parameters using video analysis is time consuming, which in turn limits its daily utilization by the coaches.

The temporal parameters (i.e., contact times, flight times and step frequency) can also be measured with a high accuracy using force platforms [2,10]. However, the step length cannot be easily measured with force platforms; this system is expensive, which also limits its daily utilization during training. Additionally, force platforms usually cover a limited area, and only a few steps can be analyzed. Hence, to overcome this limitation, another technology is broadly used for the spatiotemporal parameters’ computation: infrared mats [11]. These systems (e.g., Optojump Next) are now considered as a reference system for the computation of the spatiotemporal parameters [12] and can easily be used to investigate the spatiotemporal parameters over up to 50 m with instantaneous feedback. Yet, installing these infrared mats ahead of a training session is time consuming, and such systems cannot be used for running or sprinting events in competitions.

Hence, overcoming the above-mentioned limitations is of great interest to investigate the spatiotemporal parameters in running and sprinting both during training and competitions. With the recent technology advances with Inertial Measurement Units (IMUs), different studies attempted to compute the spatiotemporal parameters with IMUs positioned on the foot or lower back. While some studies tried to estimate the step length [13,14], most of them were interested in measuring the temporal parameters [15,16,17]. Bergamini et al. [16] and Schmidt et al. [17] demonstrated that IMUs correctly estimate the contact times and the stride duration during sprint running when data were compared to force platforms or infrared mats. More recently, Miranda-Oliveira et al. [18] developed an IMU device that can identify 88% to 98% of the number of steps and, thus, the step frequency with a high accuracy in accelerated sprinting.

Currently, Global Positioning Systems (GPSs), which are widely used in team sports, are equipped with IMUs. These GPSs were recently compared to reference systems for the velocity computation during maximal-effort 40 m sprints [19]. These authors demonstrated a high accuracy in using these GPS devices to measure the Vmax and the variables associated with the force–velocity relationship [19]. Additionally, Weber et al. [20] compared the contact times between the IMU of a GPS unit positioned close to the center of mass and a force platform. These authors reported a slight underestimation of the contact times measured with the GPS unit (−3.32%) and a large correlation coefficient (r = 0.87) between the force platforms and the GPS unit for the contact time computation [20]. Hence, through the integration of both technologies (i.e., GPS and IMU) within a single GPS unit, it is now possible to compute both the running velocity (from the GPS data) and the contact times (from the IMU data). Yet, no study to date compared the step frequency and the step length, computed from this GPS unit to a reference system. In athletics, such an opportunity would provide valuable knowledge to the coaches both during training and competitions as they are helpful for the coaches. In addition, those variables can be challenging and time consuming to evaluate, especially in competition. To date, the step frequency and step length investigation are limited to average values over 50 m based on video camera analysis [21].

Thus, the purpose of this study is to compare, from a commercial GPS unit, basic spatiotemporal parameters (step frequency and step length) during both maximal-effort sprints and steady-state running in comparison to a reference system. We hypothesized that a GPS unit, including an IMU positioned on the upper-back with appropriate data processing, would accurately yield the sprinting spatiotemporal parameters.

## 2. Experimental Design

### 2.1. Participants

Five men who are recreational runners without lower limb injuries volunteered for this study (weight: 71.63 ± 8.38 kg; height: 182.2 ± 5.7 cm). The participants were recruited for their experience in athletics. They were asked to wear personal running shoes for the test. All participants gave their informed consent for inclusion before they participated in this study. This study was conducted in accordance with the Declaration of Helsinki, and the protocol was approved by the local Research Ethics Committee prior to the initiation of this research.

### 2.2. Materials

The participants were equipped with an embedded system (Vector X7, Catapult, Melbourne, Australia) consisting of a GPS (sampled at 10 Hz) and an IMU (sampling at 100 Hz). The system was placed in a sport bra supplied by the manufacturer, allowing the system to be positioned between the shoulder blades.

The embedded systems were compared with an optoelectronic system sampling at 1000 Hz (Optojump Next, Microgate, Bolzano, Italy), considered to be a reference system. The optoelectronic system was composed of 40 individual 1 m long bars of on a standard outdoor athletic track. As the optoelectronic system allows indicators to be recorded for each step, steps were also identified on the embedded system using the vertical acceleration of the inertial unit (one step equals one wave). To ensure that the steps were correctly identified, each trial was filmed using a camera (sampling at 120 Hz, iPhone 12, Apple, Cupertino, CA, USA) and checked by an operator.

### 2.3. Procedure

After a free warm-up phase of 15 to 20 min, the participants performed six 40 m flying sprints with a 15 m approach and with their training shoes (the total distance for each sprint was then 55 m) on an outdoor track covered with tartan: (1) two sprints with running starts at “slow” velocities (S); (2) two sprints with running starts at “medium” velocities (M); and, finally, (3) two sprints with running starts at “fast” velocities (F). For each sprint, the participants started 15 m before entering the optical system. The tests were randomized, and the participants chose the velocities based on their own feelings.

### 2.4. Data Processing

To compare the two systems, only the signals from the embedded system corresponding to the steps taken in the reference system were kept. The signals were cut after observing the videos, counting the number of steps taken before entering the reference system.

Three indicators were extracted from the IMU and the GPS data and compared with the data from the reference system.

**Figure 1:**
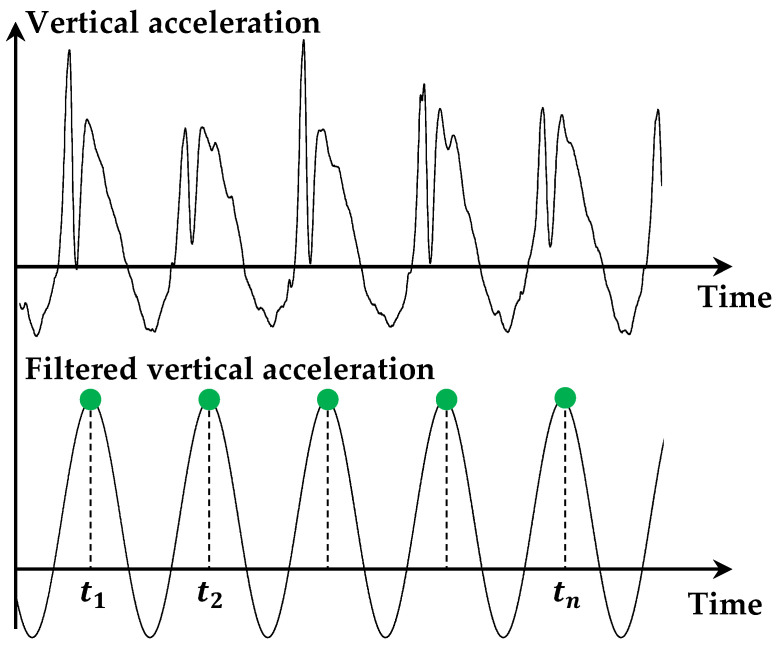
Step extraction on vertical acceleration signals. The time index of each step was deduced from the acceleration signals and used to recalculate the various indicators.

Firstly, the step frequency was calculated using the vertical acceleration measured by the IMU. The raw signal was first observed in the spectral domain using a Fast Fourier Transform. An average sprint frequency was identified as the first identifiable frequency after 0 Hz. The raw signal was then filtered using a 1st-order Butterworth bandpass filter between 0.8 and 1.5 times the average sprint frequency to clean the signal of any high-frequency components. Finally, as each step had been identified beforehand, the positive peaks of each wave/step were identified on the filtered signal (Figure 1), and the step frequency was determined as the inverse of the time between two peaks.

Secondly, each step length was calculated using the vertical acceleration measured by the IMU and the distance traveled measured by the GPS (odometer). Following the procedure used to calculate the step frequency, the positive peaks of each wave/step and their corresponding temporal indices were identified on the filtered acceleration signal. The step length was calculated as the distance measured by the GPS between two acceleration peak indices.

Finally, the step velocity was calculated directly as the average velocity measured by the GPS between the start and end of a step.

### 2.5. Statistics

The agreement between the values established by the reference system and those determined by means of the embedded system was studied by means of the coefficient of determination (r^2^) and the root mean square error (RMSE), as well as by plotting the Bland and Altman graph. For each indicator, the relative errors between the embedded system and the reference system were calculated for each step and for the sliding value of the indicators over a group of three steps.

Based on the [22] study, the classification of correlation and error was revised upwards to meet metrological performance expectations. Coefficients of determination considered very large and extremely large were associated with values greater than 0.90 and greater than 0.95, respectively. Biases were considered very low for values below 5% and low for values between 5 and 10%.

## 3. Results

For the GPS signal quality, the average horizontal dilution of precision was 0.73 ± 0.06, and the number of satellites was 16.0 ± 1.0, which lies within the upper range of good signal quality [23]. The mean velocities for the different conditions were, respectively, 4.5 +/− 0.5 m/s for S; 6.0 +/− 0.6 m/s for M; and 8.1 +/− 0.5 m/s for F. For this study, a total of 398 steps were analyzed. The relative errors are shown in Table 1. The relative errors are, on average, less than 5%.

**Figure 2:**
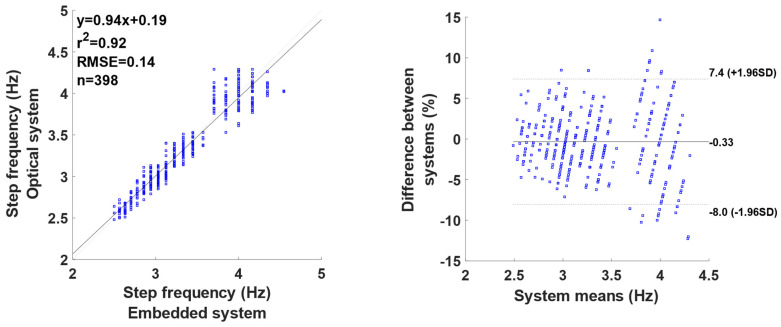
Comparison of the step frequency established between the reference system and the embedded system: correlation graph (**left**) and Bland and Altman graph (**right**).

The results for the step frequency are shown in Figure 2. The results show a very large coefficient of determination (r^2^ = 0.92) with an RMSE of 0.14 Hz. The 398 observed steps were measured between 2.5 and 4.5 Hz, with a very low bias (0.33%) and a dispersion around this bias between +7.4 and −8.0%. The results seem to show that the higher the step frequency, the greater the dispersion, a phenomenon that is generally observed at higher velocities.

**Figure 3:**
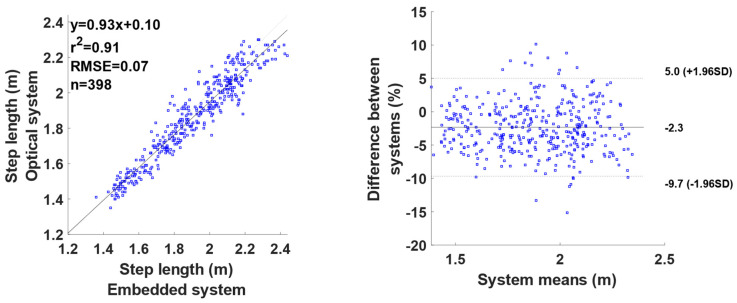
Comparison of the step length established between the reference system and the embedded system: correlation graph (**left**) and Bland and Altman graph (**right**).

The results for the step length are shown in Figure 3. The results show a very large coefficient of determination (r^2^ = 0.91) with an RMSE of 0.07 m. The 398 observed step lengths were between 1.4 and 2.4 m measured with the optical system, with a very low bias (−2.3%) and a dispersion around this bias between +5.0 and −9.7%. The results do not seem to show any trend, and the dispersion of the results seems to be random regardless of the running velocity.

**Figure 4:**
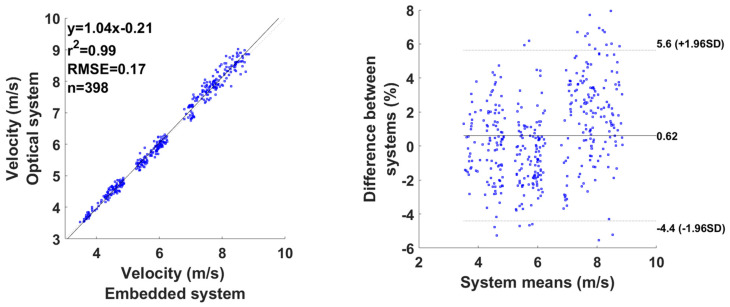
Comparison of the step velocity established between the reference system and the embedded system: correlation graph (**left**) and Bland and Altman graph (**right**).

The results for the step velocity are shown in Figure 4. The results show an extremely large coefficient of determination (r^2^ = 0.99) with an RMSE of 0.17 m/s. The 398 observed steps were between 3.5 and 9 m/s, with a very low bias (0.62%) and a dispersion around this bias between +5.6 and −4.4%. The results seem to show that the higher the step velocity, the greater the dispersion. If, visually, three velocity categories are observable on the graphs, the running velocities observed for each velocity category seem to show an overlap of data between S and M velocity.

**Figure 5:**
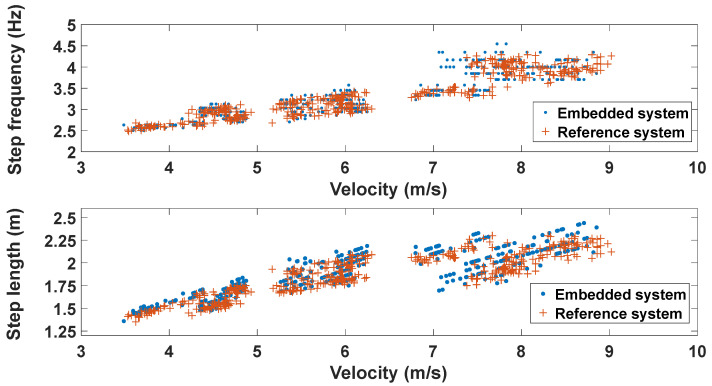
Evolution of the step frequency (**top**) and the step length (**bottom**) as a function of the velocity. The orange crosses represent the reference system, while the blue dots represent the embedded system.

Figure 5 shows the evolution of the step frequency and the step length as a function of the step velocity for the two systems. Regardless of the system, it is possible to observe an increase in the indicators with the increase in velocity, particularly between the S and the M velocities. For the F velocities, this trend seems more unpredictable, with a greater dispersion of the results.

## 4. Discussion

The present study compared the step velocity, the step frequency and the step length between a reference system (e.g., optoelectronic system) and a GPS unit integrating a GPS and a 3D IMU. Using the raw data from the accelerometer and the GPS with a customized script, we showed that we were able to identify the step frequency, the step length and the step velocity during an accelerated sprint.

### 4.1. Comparison to the Literature

The present study demonstrated that the step velocity computed using the GPS unit was close to that measured with the infrared mat (r^2^ = 0.99, RMSE = 0.17 m/s; see Figure 3). These findings support a previous study that reported variations in the Vmax of +/− 0.2 m/s between a GPS and a reference system [14]. Thus, the present work confirms that these GPS units offer accurate measurements of the running velocity when compared to an optoelectronic system.

The step frequency computed with the GPS unit is consistent with previous observations in sprinting at similar velocities [1,4]. Additionally, the step frequency showed a very large determination coefficient (r^2^ = 0.92, RMSE = 0.14 Hz; see Figure 1). The errors observed in the present study are comparable to those found in [24]’s study, which showed variations of +/−0.1 Hz. Despite the relatively low error, the data indicate an effect of the running velocity, corresponding to the highest step frequency. This effect might be firstly related to the low sampling frequency of the IMU (100 Hz) and secondly related to the algorithm’s approach of averaging the step frequency over the entire signal, without accounting for variations within the sprint. Nonetheless, the algorithm could be improved by introducing windowing for the step frequency computation.

The step length computed with the GPS unit and the custom script showed a large correlation coefficient (r^2^ = 0.91, RMSE = 0.07 m) without a velocity effect. This indicates that a change in the SL measured with the infrared mat was well measured by the GPS unit too, across different running velocities. These findings are like those of a previous study with errors around 0.08 m using embedded systems [14].

Moreover, by combining all our data of step length and step frequency at different velocities for various subjects, we can examine the effect of the step length and the step frequency on the velocity (Figure 4). The relationship between these three parameters is generally well described. Rabita et al. [2] showed that, when sprinting with maximal effort, both an increase in the step length and the step frequency led to an increasing velocity until 7 m/s. Above 7 m/s, step frequency plateaus, and the Vmax is reached by an increase in the step length [2].

Our data clearly show this typical pattern of the step length and the step frequency increase, with both the optoelectronic system and the IMU + GPS systems (Figure 4). Around 7 m·s^−1^, a plateau of the step length and an increase in the step frequency are also observed. The comparison of the step length and the step frequency obtained in the present study with the data of Dillmann [4] and Bailey et al. [25] demonstrated close values. For example, in our study, from 4 to 8 m·s^−1^, the step length and the step frequency increased from 1.45 m to 2.15 m and from 2.6 to 3.8 Hz, respectively. In Bailey et al. [25] and Dillmann [4], the step length ranged from 1.25 and 1.45 m at 4 m·s^−1^ to 2.05 to 2.15 m at 8 m·s^−1^, while the step frequency ranged between 2.75 and 3 Hz at 4 m·s^−1^ to 3.75 to 3.8 m at 8 m·s^−1^. Thus, the values we obtained in the present study are very close to those reported in these previous studies.

### 4.2. Practical Considerations

Traditionally, the assessment of sprinting and running spatiotemporal parameters has relied on optoelectronic tools or video analysis. Optoelectronic systems, while precise, are costly and necessitate the presence of a dedicated sports scientist, and the analysis is limited to a specific calibrated volume. These systems also involve extensive setup time, usage during sessions and data processing. Moreover, they are not suitable for use during competitions. On the other hand, video analysis requires space calibration for amplitude measurements and involves time-consuming data processing, typically performed over 50 m segments [21]. Therefore, the video analysis limits the ability to thoroughly analyze changes in spatiotemporal parameters throughout the entire race.

Hence, the primary advantage of the present technology lies in its ability to evaluate the spatiotemporal parameters both during training and competition, with rapid data processing. In addition, this technology allows for the assessment of parameters either at each step or averaged over multiple steps that will increase the accuracy of the measurement. Overall, it offers coaches and practitioners comprehensive spatiotemporal data over an entire sprint, training session, or season, which have been challenging to achieve with current methods.

The sprinting spatiotemporal parameters can vary up to 15% between the maximal values reached during a 400 m race and those at the end of the race [21,26]. Given the differences between our data processing and the reference system (<3.6%), we can assume that, by using the GPS unit and the present data processing, we can identify the spatiotemporal parameters changes in world-class, national and regional-level sprinters within a 400 m race. This includes comparing non-fatigued and fatigued conditions, which would help the coach and the sport practitioners to understand the underlying parameters responsible for this velocity loss.

### 4.3. Limits and Perspectives

Nevertheless, with this technology, it could be challenging to evaluate the intra-subject variation within an elite pool of athletes. Indeed, Rabita et al. [2] showed that the intra-subject coefficient of variations within a sprint session for elite athletes was 2.15% for the step length. Hence, we would postulate that this technology is not adapted, to date, to evaluate the within-session variability for elite athletes as they have a high repeatability. Another critical point could be during the start phase and the first step of the sprint. Indeed, due to the specific position of the GPS unit on the body (positioned between the shoulders), this system could not be accurate enough to measure the step frequency during the first steps. This issue could be solved if the system were placed at the hip, near the center of mass. However, this system was chosen because it is already widely used and accepted by many athletes. Placing the GPS unit closer to the legs might improve step detection [20]. In addition, the sampling rate limits the accuracy in the computation of different parameters (e.g., stance or flight times). We bet that future generations of GPS units will increase the GPS-unit sampling rate, which will undoubtedly improve the computation of the step length, the step frequency and could even make the measurement of the stance and flight times possible. The increase in the sample rate of the IMU will allow for improvements on the time detection of the peaks on the acceleration signal induced by the foot landing (Figure 1). The improvement of time detection will improve the accuracy of the computation of the stride rate, especially at a velocity higher than 7 m/s.

Another limit of the present study was the running ability of our participants. Indeed, the participants enrolled in this study were not high-level sprinters, which could also have influenced the sprint technique and eventually impacted the acceleration. Overall, the parameters derived from the GPS unit were strongly correlated with the reference system. This means that a change in the step velocity, step frequency or step length detected with the reference system was also detected well by the GPS unit.

## 5. Conclusions

The present study demonstrates that using an Inertial Measurement Unit integrated within a GPS unit allows for highly accurate identification of the sprinting spatiotemporal parameters (step length and step frequency) compared to a reference system. The innovation of the present method lies in its ability to evaluate the sprinting spatiotemporal parameters both in training and competition, with rapid data processing. This rapid evaluation provides coaches with valuable insights into the mechanical determinants of sprint performance, both during training sessions and competitive events. By overcoming the traditional challenges of a time-consuming and complex analysis, this study opens new avenues for efficiently assessing spatiotemporal parameters in diverse contexts, ultimately enhancing performance monitoring and optimization.

## Figures and Tables

**Table 1:** Relative errors for each step or group of three steps.

	Relative Errors (%) For Each Step
Step Frequency	3.08 ± 2.46
Step Length	3.55 ± 2.45
Step Velocity	2.12 ± 1.62

## Data Availability

The data presented in this study are available upon request from the corresponding author. The data are not publicly available due to privacy.
